# The influence of freeze-thaw action and particle size characteristics on the shear resistance of black soil

**DOI:** 10.1038/s41598-026-36780-z

**Published:** 2026-01-24

**Authors:** Rongfei Zhao, Haohao Chang, Jincheng Yu, Donghao Huang, Defeng Yang, Huimin Yang, Lili Zhou

**Affiliations:** 1 College of Water Conservancy, Shenyang Agricultural University, Shenyang, 110866 Liaoning China; 2 Liaoning Provincial Key Laboratory of Soil Erosion Prevention and Ecological Restoration, Shenyang, 110866 China

**Keywords:** Freeze-thaw, Particle size, Shear strength, Typical black soil in northeast china, Ecology, Ecology, Environmental sciences

## Abstract

To explore how freeze-thaw action modulates the relationship between particle size and soil shear strength, cohesion (*c*), internal friction angle (*φ*), and shear strength (*τ*) of seven particle size groups (at 4% water content) were measured using a direct shear apparatus. Results show: Particle size significantly influences *c* dynamics. naturally graded soil and d ≥ 1 mm (particle size ≥ 1 mm) groups exhibit decreasing *c* with freeze-thaw cycles, while d < 1 mm groups show the opposite trend. Among them, d_5–10_ mm groups are least affected, and d < 0.25 mm groups are most affected, with *c* stabilizing after 6–9 cycles. For *φ*, d ≥ 2 mm groups first increase then decrease, whereas d < 2 mm groups show the reverse. d < 0.25 mm groups retain the highest *φ* values; after 30 cycles, d_2 –5_ mm groups exhibit the largest *φ* decrease (− 4.70%), while d_0.5−1_ mm groups show a slight increase (2.17%). Naturally graded soil has the highest *τ* due to inter-particle synergistic effects, with d_1–2_ mm groups leading among single particle size groups. *τ* correlates positively with cycles for d < 1 mm groups but negatively for naturally graded soil and d ≥ 1 mm groups. Particle size dominates shear resistance (*c*:71.78%, *φ*:45.43%, *τ*:53.22%), with freeze-thaw cycles as a key secondary factor (18.82%, 11.27%, 20.52%).

## Introduction

As a typical environmental disturbance factor, freeze-thaw action not only exerts a pronounced effect on the mechanical properties of soil but also impacts the structural and mechanical characteristics of other materials (e.g., cement mortar) to varying degrees—this study, however, focuses on its specific mechanisms of influence on the black soil in Northeast China^1–3^. In Northeast China, driven by climatic and environmental factors, freeze-thaw action on surface soil aggregates in spring and autumn annually disrupts their stability, inducing changes in their mechanical properties and exacerbating soil erosion^4,5^. Soil shear strength is a key indicator characterizing soil mechanical properties. Clarifying the variation patterns of shear strength in soils with different particle sizes under freeze-thaw action is of great significance for studying issues such as soil erosion and water-induced soil loss^6,7^. Regarding the influence of freeze-thaw action on soil shear strength, domestic and international scholars have conducted research on various soil types, including loess^8,9^, black soil^10,11^, Qinghai-Tibet clay^12^, and soft soil^13^. Results indicate that freeze-thaw action alters soil pore structure, bulk density, and aggregate stability, thereby influencing soil shear strength. However, due to differences in soil properties and test methods, there is no consensus yet on the specific variation patterns of shear strength with freeze-thaw cycles. Zuo et al. indicated that under a certain water content, the cohesion of black soil gradually decreases with increasing freeze-thaw cycles, with one freeze-thaw cycle being the critical threshold with the most significant impact; After one cycle, soil cohesion tends to stabilize^14^. In contrast, Steiner et al. argued that one freeze-thaw cycle has no significant effect on soil shear strength and cohesion, especially when soil water content is low^15^. Fan et al.^16^ concluded that the cohesion of black soil gradually stabilized only after three freeze-thaw cycles. In addition to freeze-thaw cycles, an increase in water content also induces a sharp decrease in soil cohesion^17^. Studies on internal friction angle have also yielded conflicting conclusions. Zhang et al. conducted a study on sandy clay and found that the soil cohesion exhibits a significant decreasing trend with an increasing number of freeze-thaw cycles. After 15 freeze-thaw cycles, the cohesion can decrease by 3.81 kPa to 7.95 kPa. In contrast, the internal friction angle undergoes only a slight reduction under the influence of freeze-thaw cycles. Furthermore, the decrease in soil shear strength primarily occurs within the first 10 freeze-thaw cycles^18^. Quan et al. found that after multiple freeze-thaw cycles, soil internal friction angle first decreases and then increases^19^; Wang et al. argued that internal friction angle decreases with increasing freeze-thaw cycles^20^; however, Zhu et al. observed in their studies on brown soil that internal friction angle does not change significantly with increasing freeze-thaw cycles^21^. Liu et al. conducted consolidated direct shear tests and scouring tests on black soil in Northeast China and found that the shear strength, cohesion, and internal friction angle of black soil gradually decrease with an increasing number of freeze-thaw cycles before tending to stabilize. Furthermore, the influence of freeze-thaw cycles exhibits a cumulative effect, with a threshold value observed between 5 and 7 cycles^22^.

Freeze-thaw action causes the disruption and reformation of soil aggregates, thereby leading to changes in mechanical shear strength^23,24^. In addition, soil shear strength varies with different particle size distributions^25^. Notably, Ning et al. studied the influence of particle size on the dynamic properties of gravelly soil. The results indicated that the dynamic shear modulus $\:{\mathrm{G}}_{\mathrm{d}}$, normalized shear modulus $\:{\mathrm{G}}_{\mathrm{D}}/{\mathrm{G}}_{\mathrm{m}\mathrm{a}\mathrm{x}}$, and damping ratio $\:\lambda\:$ of gravelly soils exhibit a pronounced dependence on $\:{\mathrm{d}}_{\mathrm{m}\mathrm{a}\mathrm{x}}$. Under the same confining pressure, the maximum shear modulus $\:{\mathrm{G}}_{\mathrm{m}\mathrm{a}\mathrm{x}}\:$ increases with increasing particle size. For a given dynamic shear strain, $\:{\mathrm{G}}_{\mathrm{D}}/{\mathrm{G}}_{\mathrm{m}\mathrm{a}\mathrm{x}}$ decreases as particle size increases, whereas the damping ratio increases with particle size^26^. Yang et al. found through temperature measurement that under different negative freezing temperatures, the unfrozen water content shows an increasing trend with the increase in fine particle content. Under the condition of the same compaction degree, the ice content of soil samples subjected to different negative freezing temperatures increases as the fine particle content rises. The cohesion of the soil samples follows the same pattern as the ice content, while the internal friction angle of the soil samples decreases with the increase in fine particle content across all negative freezing temperatures tested^27^. Du et al. conducted laboratory direct shear tests on remolded soils with different coarse particle contents and found that an increase in coarse particle content reduced soil volumetric shear shrinkage deformation and cohesion, while increasing the internal friction angle^28^. Yu et al.^29^ mixed three types of soil with different particle sizes with soil particles smaller than 0.075 mm. Their results showed that with increasing coarse-grained soil content, both the shear strength and internal friction angle of the mixed soil samples increase. Li et al. conducted direct shear tests on coarse-grained soil. The experimental results showed that the relationship between moisture content and shear strength in coarse-grained soil is complex and non-linear. With other parameters held constant, increasing moisture content from 2 to 12% resulted in a progressive decrease in internal friction angle (up to 26.91%), while cohesion exhibited an initial increase (up to 44.27% at 8% moisture content) followed by a decrease (up to 16.93%). This non-monotonic behavior underscores the critical role of moisture in soil strength dynamics. However, most existing studies only consider the influence of particle size on soil shear strength under non-freeze-thaw conditions, leaving a research gap regarding how particle size affects soil shear strength under freeze-thaw action^30^.

To further clarify the mechanisms by which freeze-thaw action and particle size characteristics influence soil shear strength, this study conducted laboratory freeze-thaw cycle tests and direct shear tests on typical black soil from Northeast China. It investigates the relationship between freeze-thaw action, soil particle size characteristics, and shear strength, and gains a deeper understanding of how these factors affect the shear strength of typical black soil. These efforts aim to clarify the variation patterns of soil mechanical properties under freeze-thaw conditions, providing a scientific basis for the rational protection and utilization of typical black soil. This research thus holds great significance for providing theoretical support for the prevention and control of soil erosion in black soil regions.

## Materials and methods

### Collection and preparation of soil samples

Soil sampling was conducted in Heshan Farm, Nenjiang City, Heilongjiang Province. It is situated in the core of the Northeast Black Soil Region. The soil parent material is primarily Quaternary pluvial-alluvial deposits, with black soil as the dominant type. The main crops grown here are barley, wheat, soybeans, and corn. The collected soil samples were preliminarily processed at the Water Conservancy Experiment Base of Shenyang Agricultural University. Impurities such as plant roots and gravel were removed from the samples, which were then sieved through a 10 mm sieve. The soil was placed outdoors (at a temperature of 25 ± 5 °C) and air-dried naturally until the water content reached 4%, then reserved as naturally graded soil for later use. This study adopts an initial water content of 4%, which is representative of the natural dry state of black soil in Northeast China during winter. Being lower than the threshold water content for frost heave (approximately 10%), this value can effectively inhibit volume expansion induced by ice-water phase transition, ensuring that the effects of freeze-thaw cycles on soil mechanical properties are primarily attributed to particle rearrangement and fragmentation^31^. Additionally, all experiments were conducted under this consistent water content condition to maintain uniformity. Under the condition of 4% initial water content, the dry density of the black soil ranges from 1.54 to 1.60 g/cm³. In this study, the in-situ dry density was determined via the cutting ring method to be 1.57 ± 0.03 g/cm³.The retained soil was successively sieved through standard sieves of 5 mm, 2 mm, 1 mm, 0.5 mm, and 0.25 mm. Soil fractions of different particle sizes(naturally graded soil, d_5–10 mm_, d_2–5 mm_, d_1–2 mm_, d_0.5−1 mm_, d_0.25−0.5 mm_, d < 0.25 mm) were separately loaded into ring knives with an inner diameter of 61.8 mm and a height of 20 mm and wrapped with plastic wrap for later use. Meanwhile, the naturally graded soil that did not pass the standard sieves (d ≤ 10 mm) was used as the control group. The basic physical and chemical properties of the soil are presented in Table 1.

**Table 1:** Basic physical and chemical properties of soil.

Agrotype	Bulk density /(g cm^−3^)	Organic matter/(g kg^−1^)	pH	Soil mechanical composition(%)		
				Clay	Silt	Sand
Black soil	1.2	25.53	6.5	9.42	71.38	19.20

### Freeze-thaw conditions

The freeze-thaw cycle process of soil samples was carried out in a low-temperature cyclic test chamber (Model AW6000, Shenyang Yileng Refrigeration Machine Co., LTD.). The cooling method was air cooling, with a temperature range of − 30 °C to 30 °C ± 2 °C. Both the heating and cooling rates were set at 3 °C min^−1^.The freezing temperature was set at − 8 °C for 12 h, and the thawing temperature at 10 °C for 12 h. This setting is based on the consensus that after 12 h of continuous freeze-thaw exposure, the response of soil structure and deformation characteristics to freeze-thaw effects reach a stable level^32^. This temperature regime was established based on the fact that the black soil region of Northeast China features a temperate continental monsoon climate, where the minimum winter temperature typically ranges from − 10 °C to − 5 °C, and the maximum surface temperature after spring snowmelt can reach 10 °C to 12 °C^33^. Specifically, − 8 °C represents the typical lower limit of negative temperatures in this region, ensuring the complete freezing of all pore water within the soil; conversely, 10 °C corresponds to the upper limit of natural thawing temperatures in spring, enabling rapid and thorough melting. After 16 freeze-thaw cycles, the soil mass undergoes a process similar to material fatigue. Multiple freeze-thaw cycles are equivalent to applying multiple cyclic loads to the soil mass, causing it to reach a fatigue state. More freeze-thaw cycles no longer have a significant impact on it, and the internal structure of the soil sample reaches a new dynamic equilibrium^34^. Based on this, an upper limit of 30 cycles was established to cover the maximum extreme annual cycles in the region^35^. The number of freeze-thaw cycles was set as 0, 1, 2, 3, 4, 5, 6, 9, 15, 20, and 30, resulting in 11 experimental groups. The detailed experimental design is presented in Table 2.

**Table 2:** Experimental design of freeze-thaw cycles.

Soil particle size /mm	Initial water content/%	Number of freeze-thaw cycles/times	Freezing temperature/°C	Melting temperature/°C
naturally graded soil	4	0, 1, 2, 3, 4, 5, 6, 9, 15, 20, 30	− 8	10
5–10	4	0, 1, 2, 3, 4, 5, 6, 9, 15, 20, 30	− 8	10
2–5	4	0, 1, 2, 3, 4, 5, 6, 9, 15, 20, 30	− 8	10
1–2	4	0, 1, 2, 3, 4, 5, 6, 9, 15, 20, 30	− 8	10
0.5-1	4	0, 1, 2, 3, 4, 5, 6, 9, 15, 20, 30	− 8	10
0.25–0.5	4	0, 1, 2, 3, 4, 5, 6, 9, 15, 20, 30	− 8	10
< 0.25	4	0, 1, 2, 3, 4, 5, 6, 9, 15, 20, 30	− 8	10

### Shear strength determination

A strain-controlled direct shear apparatus (Model ZJ, Nanjing Soil Instrument Factory) was used to perform quick shear tests in accordance with GB/T 50,123-2019 Standard for Soil Test Methods (2019)^36^. The direct shear tests were conducted at a shear rate of 0.8 mm/min, with four sets of normal stresses applied: 100, 200, 300, and 400 kPa. Each test group was replicated three times. Soil cohesion and internal friction angle were calculated using Eqs. (1) and (2), respectively:

1 $$\:\tau\:=10{CR}/{A}$$

2 $$\:\tau\:=c+\sigma\:\mathrm{t}\mathrm{a}\mathrm{n}{\upphi\:}$$

In the equations: *τ* is soil shear strength (kPa); *R* is the reading of the force ring gauge (0.01 mm); *C* is the force ring calibration coefficient (1.88 mm/min); *A* is the stress-bearing area of the sample (cm^2^); *c* is soil cohesion (kPa); *σ* is normal stress (kPa); *φ* is soil internal friction angle (°).

The weighted average soil shear strength (*τ*_*0*_) was calculated using Eq. (3):

3 $$\:{\tau\:}_{0}=\left({\sum\:}_{\mathrm{i}=1}^{4}{\sigma\:}_{\mathrm{i}\:}{\tau\:}_{\mathrm{i}}\right)/\left({\sum\:}_{i=1}^{4}{\sigma\:}_{i\:}\right)$$

In the formula: *τ*_*0*_ is the average soil shear strength (kPa); *σ* is the normal stress (kPa).

### Data analysis

IBM SPSS Statistics 25.0 was used for one-way and two-way analysis of variance (ANOVA), while Origin 2022 was employed for regression analysis and plotting.

Based on the ANOVA results, the relative contributions of freeze-thaw cycle number, particle size characteristics, and their interaction to cohesion, internal friction angle, and average shear strength were calculated using Eq. (4)^37^, Results are presented in Table 3.

4 $$\:CT=(SS-MSE\times\:\:df)/SST$$

In the formula: *CT* is the contribution degree (%); *SS* is the sum of squares; *MSE* is the mean square error, *df* is the degrees of freedom, *SST* is the total sum of squares.

## Results and analysis

### The influence of freeze-thaw cycles and particle size characteristics on soil cohesion

**Fig. 1:**
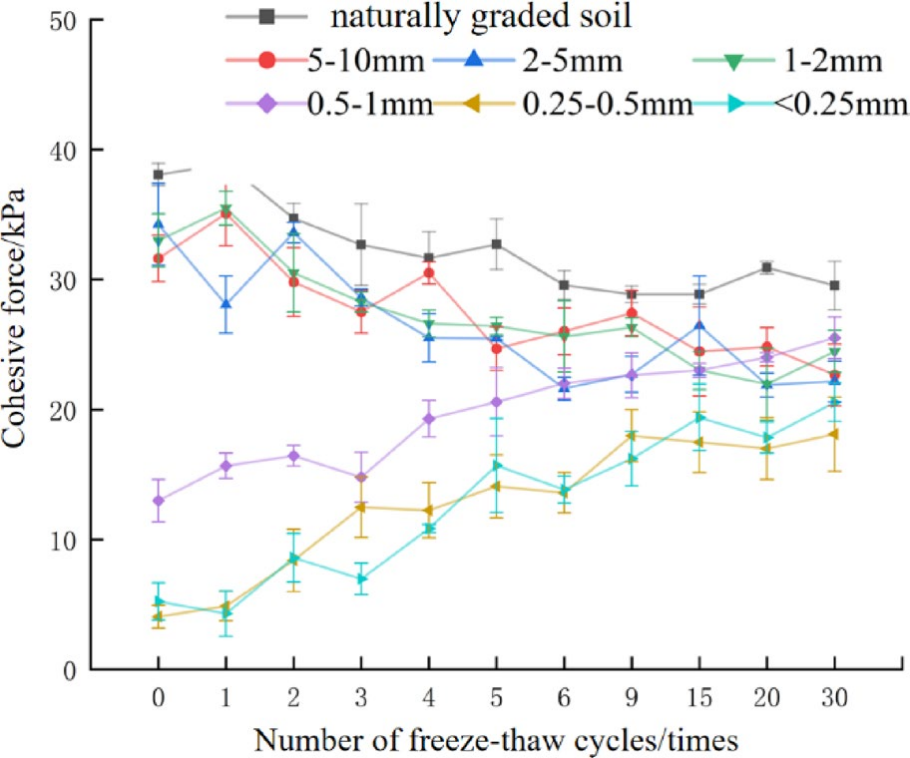
Soil cohesion of each particle size group under freeze-thaw cycle.

Soil cohesion is widely recognized as a key indicator characterizing soil shear strength, arising primarily from interparticle bonding forces and intermolecular van der Waals forces, and an important indicator reflecting soil stability and erosion resistance^38^. The changes in soil cohesion under different particle size fractions over 0–30 freeze-thaw cycles are presented in Fig. 1.

Overall, freeze-thaw action has a significant impact on soil cohesion (R^2^ > 0.51, indicating good model fit). One-way ANOVA results indicated that after 6 to 9 freeze-thaw cycles, soil cohesion across different particle size characteristics tended to stabilize. Two distinct freeze-thaw influence patterns emerged among particle size groups. For naturally graded soil and d ≥ 1 mm groups, cohesion decreased with increasing freeze-thaw cycles. After 30 cycles, cohesion in naturally graded soil, d_5–10_ mm, d_2–5_ mm, and d_1–2_ mm soil decreased by 35.6%, 28.35%, 35.29%, and 25.91% respectively relative to the non-freeze-thaw control. In contrast, cohesion in d < 1 mm soil increased with more freeze-thaw cycles: d_0.5–1_ mm, d_0.25–0.5_ mm, and d < 0.25 mm soil showed increases of 96.15%, 341%, and 292% respectively after 30 cycles. Li found that the influence of freeze-thaw on cohesion decreases with increasing particle size: coarse-grained soil (d ≥ 1 mm) exhibited smaller cohesion changes, while fine-grained soil (d<1 mm) showed larger changes^39^. Freeze-thaw cycles alter original interparticle bonds: fine particles undergo freeze-thaw-induced consolidation, enhancing cohesion, whereas bonds between coarse particles are disrupted, reducing cohesion. Across all groups, naturally graded soil had the highest cohesion both before and after freeze-thaw: 38.73 kPa pre-treatment and 30.92 kPa after 30 cycles. The lowest cohesion was observed in the d_0.25–0.5_ mm group: 4.07 kPa pre-treatment and 18.12 kPa after 30 cycles.

**Fig. 2:**
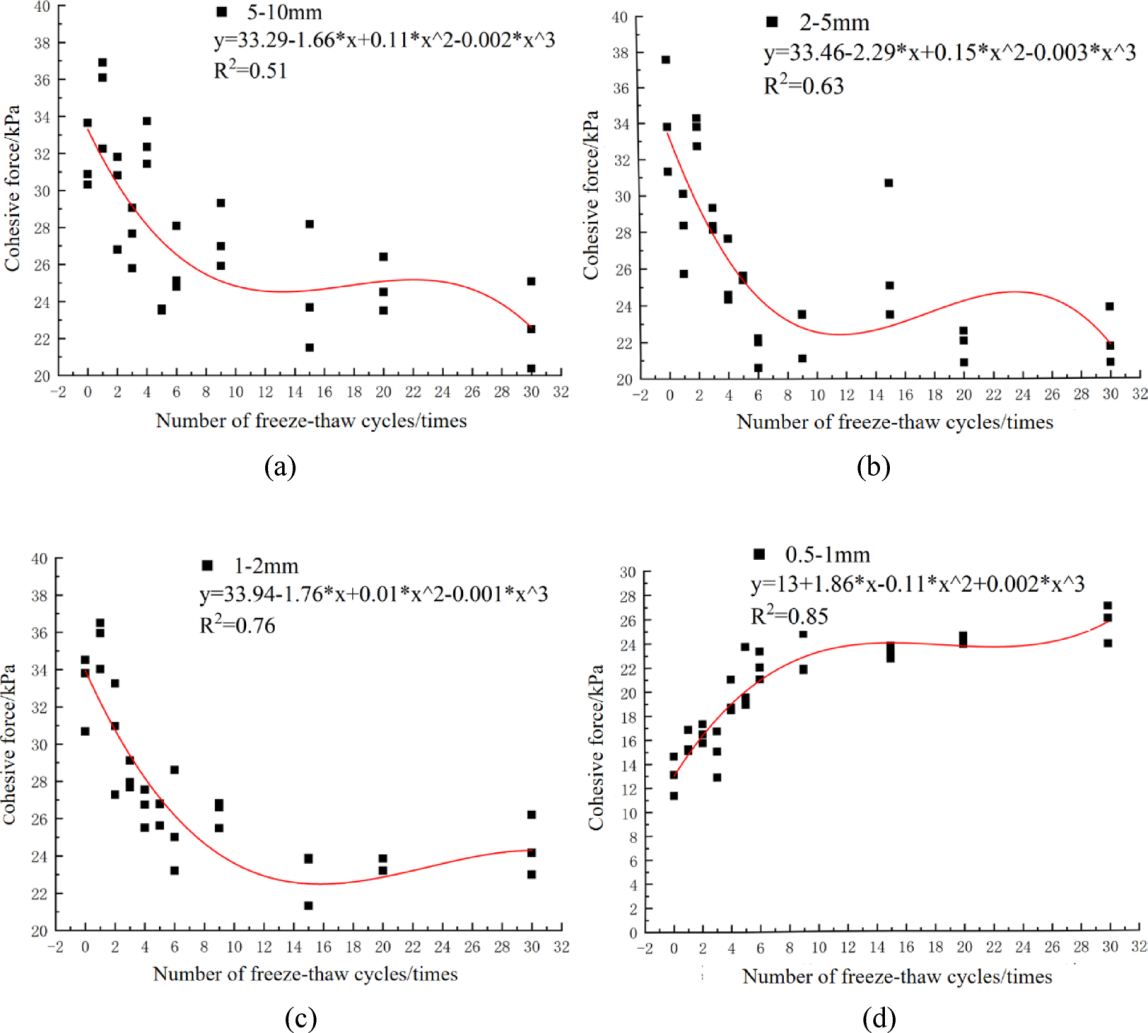

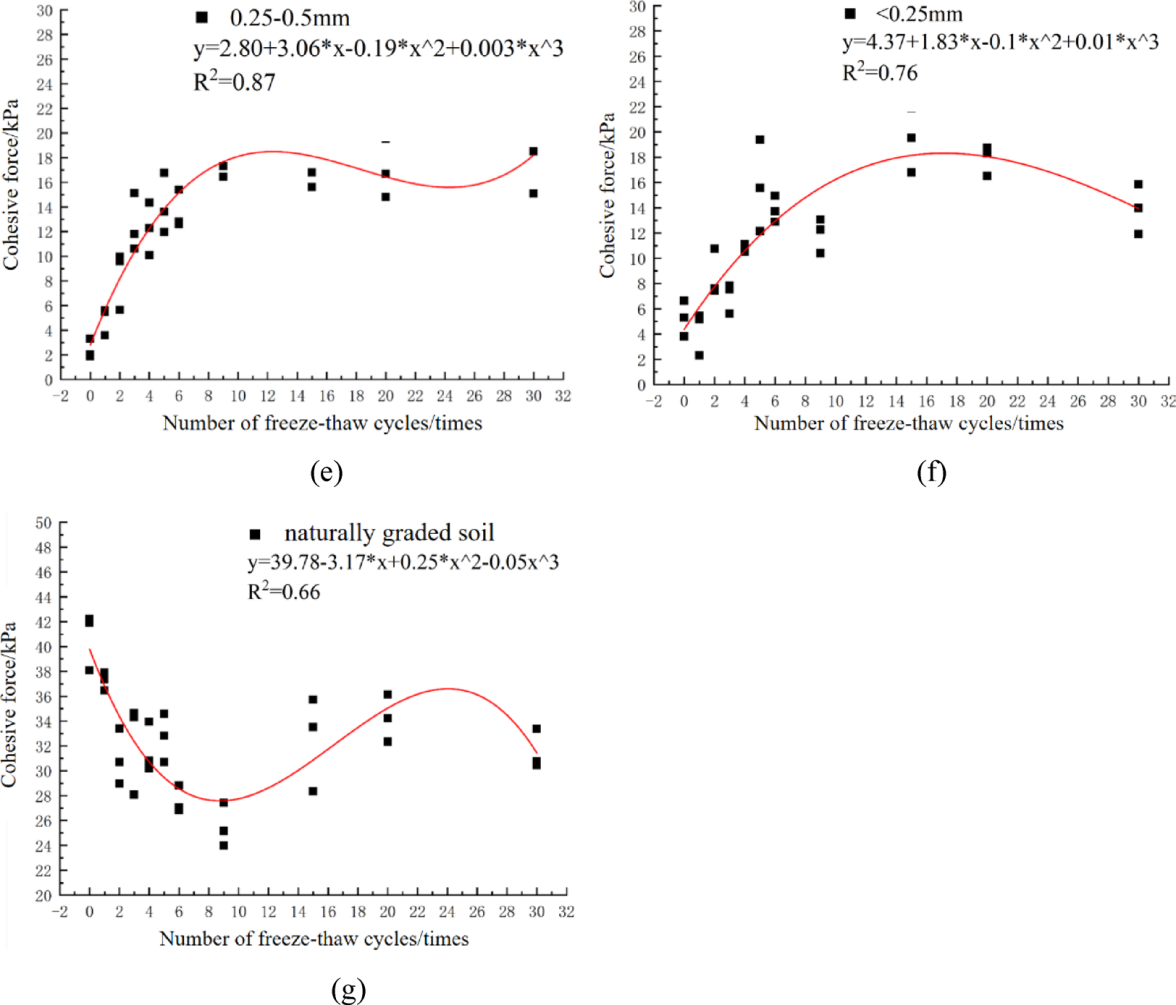
Fitting curves of soil cohesion with different particle sizes as a function of freeze-thaw times.

The variation of soil cohesion with freeze-thaw cycles in each particle size group conforms to cubic polynomial characteristics, as presented in Fig. 2.

For soil with different particle size characteristics, the cohesion curve showed an inflection point after 9 freeze-thaw cycles, marking a shift in cohesion variation trends. After 9 cycles, the slope of the curve decreased, indicating that the influence of freeze-thaw action on soil cohesion weakened in magnitude. Before the ninth freeze-thaw cycle, freeze-thaw action was the primary driver of cohesion variation; after 9 cycles, particle size characteristics emerged as the main influencing factor.

### The influence of freeze-thaw cycles and particle size characteristics on the internal friction angle of soil

**Fig. 3:**
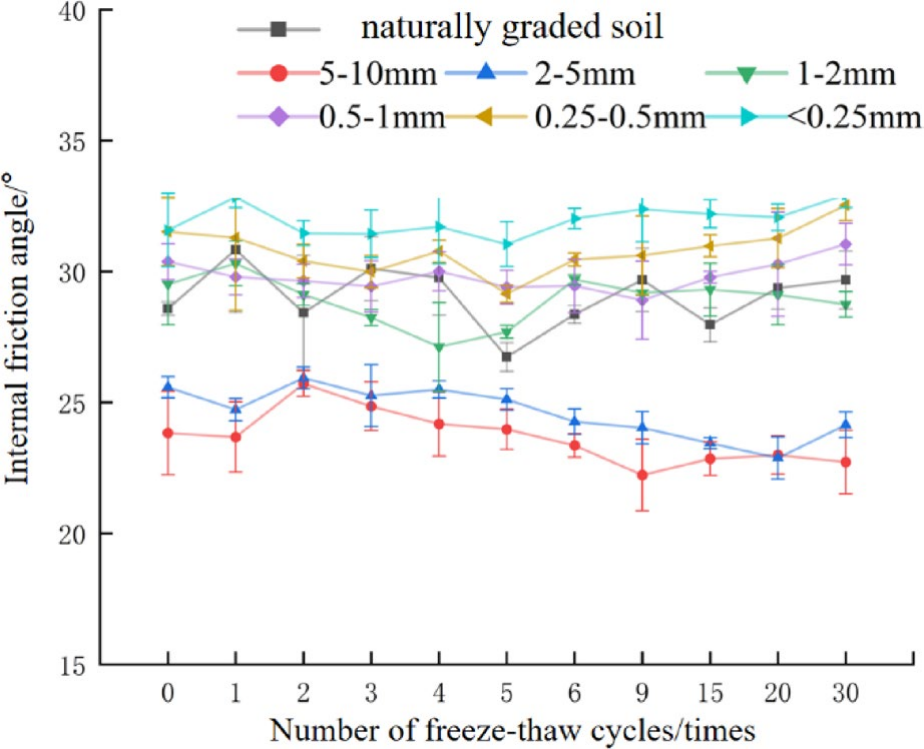
Soil internal friction angle of each particle size group under freeze-thaw cycle.

Freeze-thaw action exerted a certain influence on the internal friction angle of soils with different particle size characteristics, and the specific variation patterns are presented in Fig. 3.

When there is no freeze-thaw effect, the internal friction angle of d < 0.25 mm soil is the largest, and that of d_5–10_ mm soil the smallest. With increasing freeze-thaw cycles, the internal friction angle of the d < 0.25 mm soil remains the largest; after 30 cycles, it increased by 4.1% compared with non-freeze-thaw soil. Except for the d < 0.25 mm soil, the internal friction angles of naturally graded soil, d_0.25–0.5_ mm, and d_0.5–1_ mm soil all increased marginally after 30 cycles, whereas those of other particle size groups decreased slightly. This phenomenon arises because freeze-thaw action alters soil particle arrangement, making fine-grained soils more densely packed^19^. The internal friction angles of different particle size groups exhibit statistically significant differences. Overall, the smaller the particle size, the larger the internal friction angle. Under non-freeze-thaw conditions, d < 0.25 mm soil had the largest internal friction angle (31.58°), while d_5–10_ mm soil had the smallest (23.84°). After the first freeze-thaw cycle, the internal friction angles of d_5–10_ mm and d_2–5_ mm soil decreased by 0.06% and 3.22% respectively. Notably, compared with uniformly graded soils, naturally graded soil showed more significant variations in internal friction angle with increasing freeze-thaw cycles. This may be attributed to the fact that in mixed-particle-size soils, particles of different sizes respond to freeze-thaw processes differently. Interparticle interactions and irregular packing arrangements in mixed soils render the internal friction angle more susceptible to freeze-thaw effects.

### The influence of freeze-thaw cycles and particle size characteristics on soil shear strength

**Fig. 4:**
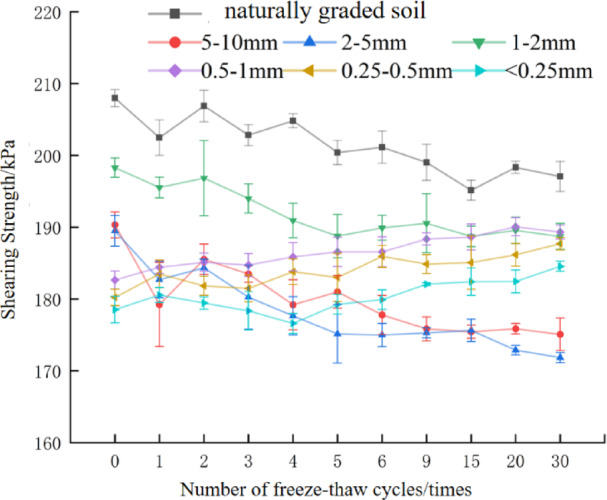
Changes of soil shear strength with the number of freeze-thaw cycles.

In the black soil region of Northeast China, soils undergo frequent freeze-thaw cycles during the annual snowmelt season; these cycles diminish soil shear strength and exacerbate soil erosion^40^. The specific variations in soil shear strength with increasing freeze-thaw cycles are presented in Fig. 4.

As can be seen from Fig. 4, similar to the variation pattern of cohesion, the shear strength of soils with particle size d ≥ 1 mm soil generally exhibited a downward trend as the number of freeze-thaw cycles increased. After 30 freeze-thaw cycles, the shear strengths of naturally graded soil, d_5–10_ mm, d_2–5_ mm, and d_1–2_ mm soil decreased by 5.32%, 8.01%, 9.31%, and 4.84% respectively relative to unfrozen soil. Among these, d_2–5_ mm soil showed the largest shear strength reduction, while d_1–2_ mm soil showed the smallest. Notably, the first freeze-thaw cycle exerted the most significant damaging effect on the soil: the reduction in shear strength after the first cycle accounted for 25.32%, 73.33%, 41.17%, and 30.3% of the total shear strength reduction after 30 cycles for these four particle size groups, respectively. This result is consistent with the findings of Zuo et al. The shear strength of soils with particle size d < 1 mm soil showed an increasing trend as the number of freeze-thaw cycles increases. After 30 freeze-thaw cycles, the shear strengths of d_0.5−1_, d_0.25−0.5_, and d < 0.25 soil increased by 3.70%, 4.00%, and 3.40% respectively compared with those of unfrozen soil.

## Discussion

The experimental results of this study are based on air-dried soil with a water content of approximately 4%, as water content is a key factor influencing soil cohesion and internal friction angle. Sun et al. conducted experiments under low-temperature and variable water content conditions. At a water content of 0%, the soil cohesion reached 220 kPa; as the water content increased to 4%, it remained at a relatively high level (> 180 kPa), while the internal friction angle exhibited a relatively small variation with changes in water content^41^. Li et al.’s experimental study on unsaturated expansive soils revealed that when the initial water content approaches the soil’s plastic limit (approximately 4%), the cohesion reaches its peak value while the internal friction angle remains at a relatively high level; a further increase in water content results in a significant attenuation of cohesion and a decrease in the internal friction angle^42^.

### The influence of freeze-thaw cycles on the shear resistance of soil

As can be seen from Table 3, the contributions of freeze-thaw cycles to variations in soil shear strength, internal friction angle, and cohesion are 18.82%, 11.27%, and 20.52% respectively. Mechanistically, during freeze-thaw cycles, pore water within the soil matrix freezes and expands, causing both increased gaps between soil particles and decreased contact surfaces between particles. When the ice melts, these expanded pores drive soil rearrangement and restructuring, leading to soil loosening and interparticle relative movement, thereby reducing the shear strength of coarse-grained soil (d ≥ 1 mm)^43,44^. Furthermore, Steiner et al. demonstrated that fine-grained soil, owing to its smaller particle size, relatively uniform particle structure, and reduced interparticle relative displacement, exhibits greater resistance to deformation and damage caused by freeze-thaw cycles^15^. Therefore, the shear strength of coarse-grained soil decreases after freeze-thaw cycles, mainly due to interparticle relative movement and structural loosening of the soil matrix, while that of fine-grained soil (d < 1 mm) increases, primarily attributed to the interlocking effect between soil particles and relative structural stability. In this study, two distinct trends in shear strength were observed for soils categorized by different particle sizes as the number of freeze-thaw cycles increased: soils with particle size d < 1 mm soil showed an overall increasing trend in shear strength, while those with coarser particle sizes exhibited a decreasing trend. This is consistent with the research findings of Adeli et al.^45^. As the number of freeze-thaw cycles increases, the cohesion of soils with d ≥ 1 mm soil generally shows a decreasing trend, while that of soils with d < 1 mm soil exhibits a gradual increasing trend. This is because freeze-thaw cycles induce the expansion of micro-pores between larger soil particles, leading to the loosening of the internal structure of the soil and thereby reducing its overall shear strength. Conversely, cohesion between smaller soil particles increases, thereby enhancing the soil’s shear strength^46,47^.

The stability of the internal friction angle of soil depends on the particle size characteristics of the soil, specifically changes in soil particle shape, sizes, and structure. From the perspective of soil particle size influence: as the number of freeze-thaw cycles increases, soil particles become more uniform. Fine soil particles aggregate into larger ones due to extrusion between ice crystals or soil aggregates, reducing specific surface area and interparticle contact points and thus decreasing the internal friction angle. Conversely, larger soil particles break down into smaller ones, increasing specific surface area and contact points and thereby raising the internal friction angle. The magnitude of these changes in soil structure is weakened^48^.

**Table 3:** The contribution of freeze-thaw times and particle size to each index.

Factors	Cohesion/kPa		Internal friction angle/°		Shear strength/kPa	
	*P*	CT (%)	*P*	CT (%)	*P*	CT (%)
Freeze-thaw times	< 0.01	20.52	< 0.01	11.27	< 0.01	18.82
Particle size	< 0.01	52.33	< 0.01	45.43	< 0.01	71.78
Freeze-thaw times× Particle size	< 0.01	23.76	< 0.01	33.83	< 0.01	5.07
Error		3.39		9.47		4.95
Principal factor sum		72.85		56.7		90.6

Note: *P* < 0.05 indicates that the data is statistically significant.

### The influence of particle size characteristics on soil shear resistance

**Fig. 5:**
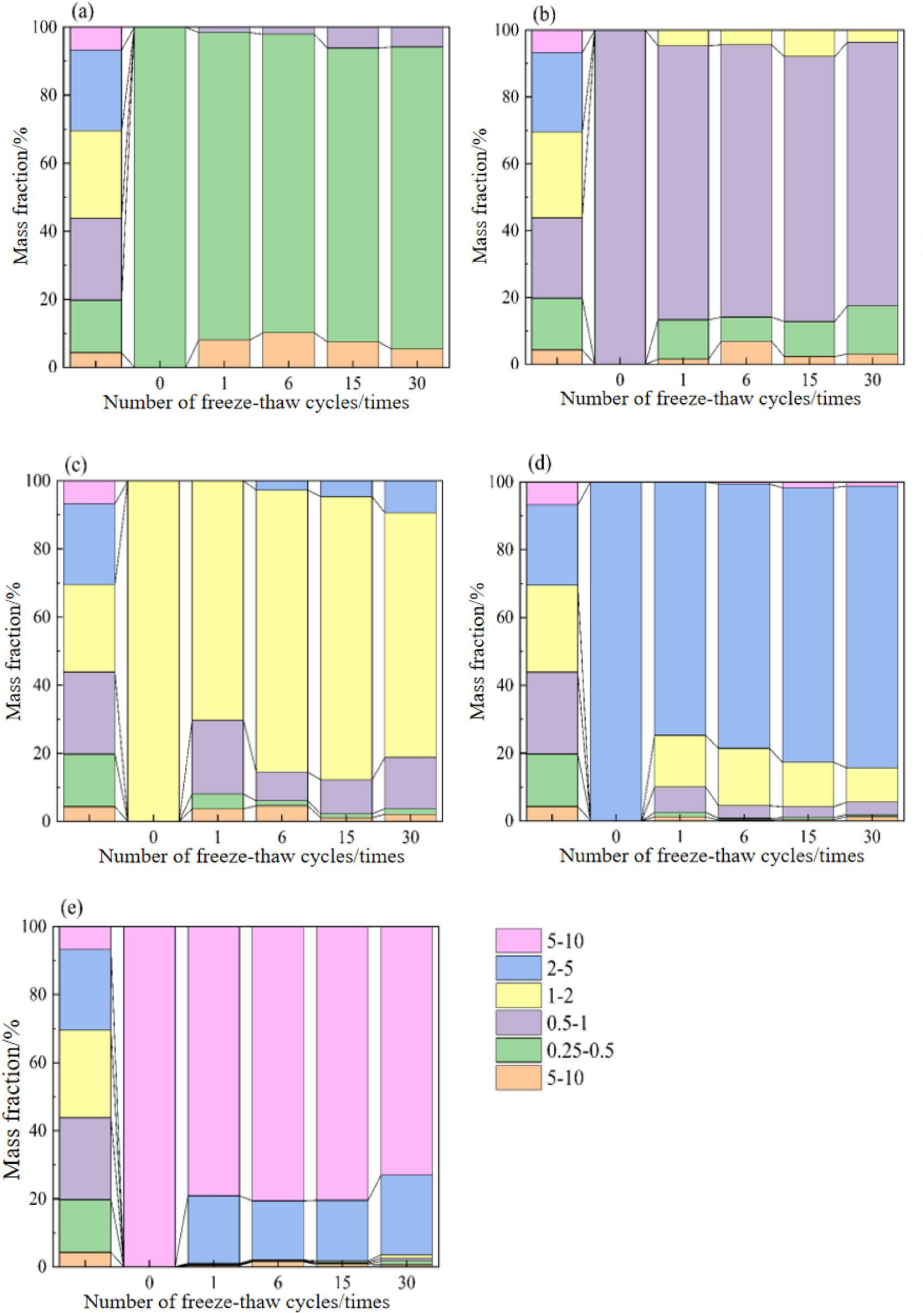
Changes in soil particle size and mass fraction under freeze-thaw action. (**a**) 0.25–0.5 mm, (**b**) 0.5–1 mm, (**c**) 1–2 mm, (**d**) 2–5 mm, (**e**) 5–10 mm.

As can be seen from Table 3, the contributions of particle size characteristics to variations in soil shear strength, internal friction angle, and cohesion are 71.78%, 45.43%, and 53.22% respectively. Notably, soils with different particle size characteristics exhibit distinct shear resistance performance after freeze-thaw cycles^49^. The freeze-thaw process exerts a dual effect on soil particles: on one hand, it fragments larger particles in the soil into smaller ones; on the other hand, it induces smaller particles to aggregate into larger ones^50^. Figure 5 shows the changes in the mass fraction of soils across various particle size ranges under freeze-thaw action, especially the changes in mass fractions for each particle size before freeze-thaw cycles and after 1, 6, 15, and 30 cycles. Quantitative data from the table indicates that freeze-thaw cycles drive changes in particle size distribution, which is likely attributed to the fragmentation of larger particles during freeze-thaw cycling. During this process, the mass fraction of d_2 − 5_ mm soil remains relatively stable with minimal variation, indicating that soil particles within this range respond relatively slowly and stably to freeze-thaw cycles. The mass fraction of d_1 − 2_ mm soil shows significant variations during freeze-thaw cycles, with a gradual shift toward d_0.5−1_ mm soil. Correspondingly, the mass fraction of particles in the d_0.5−1_ mm range increases notably after freeze-thaw cycles, indicating that d_0.5−1_ mm soil may play a major regulatory role during the freeze-thaw process. Freeze-thaw cycles may make it easier for particles within this size range to move or redistribute. During freeze-thaw cycles, the mass fraction of d_0.25−0.5_ mm soil particle size range exhibited a significant increase, particularly as the number of freeze-thaw cycles increases. This reflects the special response of d_0.25−0.5_ mm soil particle size range during the freeze-thaw process. By cross-referencing data from Figs. 1 and 3, and 5, it is evident that the influence of freeze-thaw action on soil shear resistance is mediated by changing the particle size and spatial arrangement of particles, thereby influencing its cohesion and internal friction angle.

It should be noted that the explanation of soil mechanical degradation mechanisms presented in this study remains a hypothetical analysis, as it has not yet been supported by direct evidence from microstructural observation or pore size measurement. The rationality of this mechanism requires further validation in subsequent research through micro characterization techniques, which will involve observing changes in soil particle arrangement, pore morphology, and pore size distribution.

### The interactive effect of freeze-thaw cycles and particle size characteristics

With the number of freeze-thaw cycles and soil particle size (median particle size adopted, excluding naturally graded soil samples) as independent variables, and soil cohesion and internal friction angle as response variables, analysis of variance (ANOVA) for regression equations was conducted using Design-Expert 13 software^51^. Subsequently, regression models were developed to describe the relationships among the number of freeze-thaw cycles (x₁), soil particle size (x_2_), soil cohesion (*c*), and internal friction angle (*φ*). The regression equations are as follows:

5 $$\:c=10.09+0.38{x}_{1}+8.19{x}_{2}-0.1{x}_{1}{x}_{2}-0.003{x}_{1}^{2}-0.72{x}_{2}^{2}$$

6 $$\:\phi\:=32.2-0.07{x}_{1}-2.78{x}_{2}-0.02{x}_{1}{x}_{2}+0.004{x}_{1}^{2}+0.24{x}_{2}^{2}$$

**Fig. 6:**
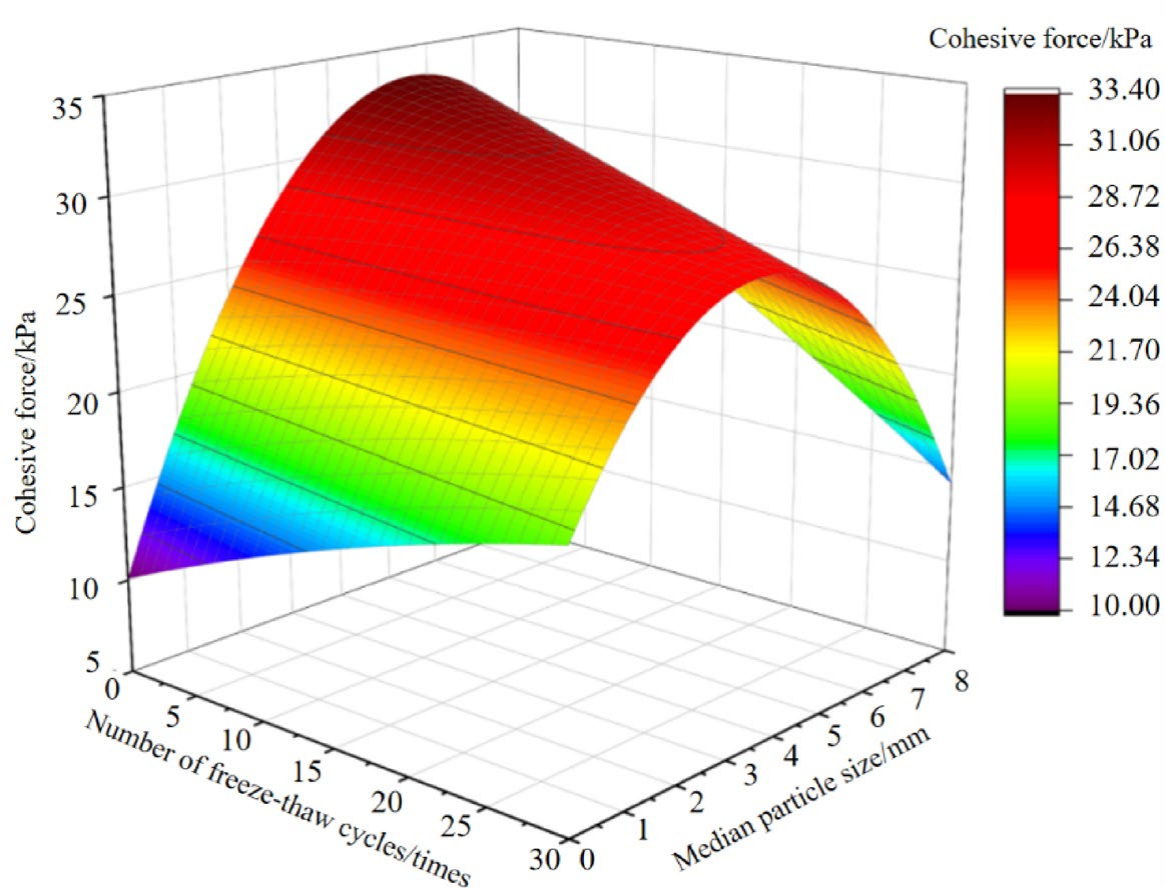
Response surface of soil cohesion to the number of freeze-thaw cycles and median particle size.

**Fig. 7:**
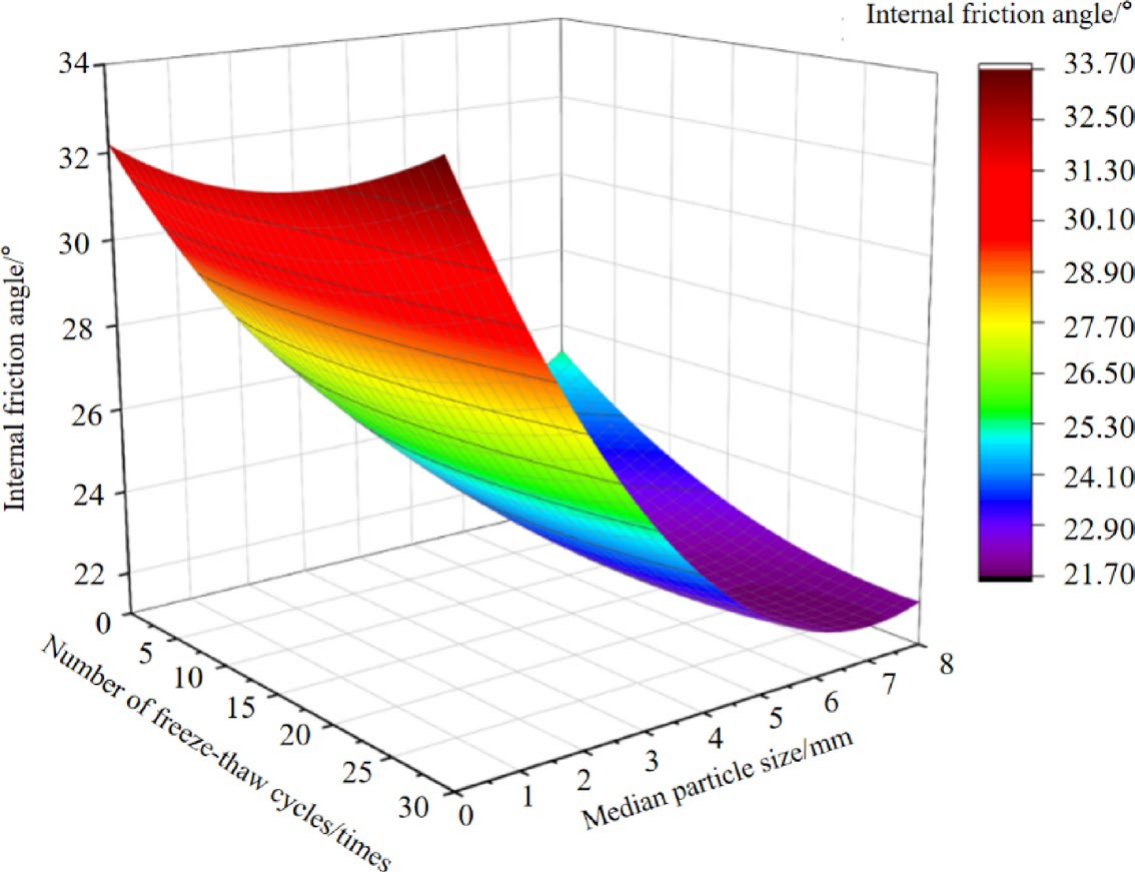
Response surface of the friction angle in the soil to the number of freeze-thaw cycles and the median particle size.

For regression model Eq. (5), the p-value is < 0.05 (indicating statistical significance) with a coefficient of determination (R²) of 0.65; for regression model Eq. (6), the p-value is < 0.05 (statistically significant) and the coefficient of determination (R²) is 0.95. Both regression models exhibit good fit, with Eq. (6) showing stronger explanatory power. Response surfaces illustrate the relationships between soil cohesion, internal friction angle, number of freeze-thaw cycles, and soil particle size, derived from the regression equations, are shown in Figs. 6 and 7. The steepness of the response surface reflects the degree of variation of cohesion and internal friction angle with the number of freeze-thaw cycles and particle size, respectively. The steeper the curve, the more significant the impact. Specifically, as can be seen from Fig. 6, for soils that have not undergone freeze-thaw treatment, soil cohesion gradually increases with increasing particle size. Following 30 freeze-thaw cycles, as particle size increases, soil cohesion first increases and then decreases. d_2–5_ mm soil exhibits the least change in cohesion, indicating that soils within this particle size range have the strongest ability to resist freeze-thaw-induced erosion and structural damage. With increasing freeze-thaw cycles, the cohesion of soils with different particle size characteristics exhibits two distinct trends: increasing and decreasing, while the cohesion difference among them gradually narrows. Notably, the curve along the particle size direction becomes significantly steeper, indicating that after freeze-thaw action, the influence of particle size on soil cohesion is greater than that of the number of freeze-thaw cycles on post-freeze-thaw soil cohesion.

As can be seen from Fig. 7, with increasing particle size, The internal friction angle of soil generally exhibits a decreasing trend. When d > 2 mm soil, the influence of freeze-thaw action on the internal friction angle is greater than that of the particle size itself. In contrast, when d < 2 mm soil, the influence of freeze-thaw action is smaller than that of particle size itself.

Both freeze-thaw action and particle size characteristics influence soil cohesion and internal friction angle, with the influence of particle size characteristics exceeding that of freeze-thaw action. This indicates that particle size characteristics are the primary factor governing these mechanical properties.

### The potential influence of initial water content on the test results

Initial water content is a key state variable governing the response to freeze-thaw cycles. A higher water content reduces the compression index, rendering fine particles more susceptible to fragmentation during freeze-thaw processes and causing the 1 mm critical threshold to shift toward finer particle sizes^31^. Increased water content (e.g., 8%–12%): A rise in water content increases the free water and capillary water within the soil. During freezing, more ice crystals form, and the volume expansion induced by the ice-water phase transition causes rapid water migration between pores, thereby intensifying the frost heave-thaw settlement process^52^. The combination of low water content and low compactness adopted in this study can maximize the retention of mechanical characteristics associated with particle rearrangement and fragmentation, rendering the 1 mm threshold representative under the experimental conditions of this research.

## Conclusion

Based on air-dried black soil with a water content of approximately 4%, this study investigates the variations in shear strength of black soil with different particle sizes under varying numbers of freeze-thaw cycles. The main conclusions are drawn as follows:

1. Freeze-thaw cycles and soil particle size characteristics have a significant influence on soil cohesion and the internal friction angle. There are differences in the responses of cohesion and internal friction angle to particle size: the sensitive particle size threshold for cohesion is 1 mm, while that for internal friction angle is 2 mm. In terms of the comprehensive contribution to shear strength, cohesion dominates the shear characteristics of black soil (especially under freeze-thaw conditions with low water content), and the bonding effect exerts a more significant influence on strength^14^. When the critical threshold particle size is 1 mm, the cohesion and internal friction angle of (d > 1 mm) soil tend to decrease with increasing freeze-thaw cycles. In contrast, (d < 1 mm) soil exhibit an increasing trend in both parameters.
2. Freeze-thaw cycles and particle size characteristics affect soil cohesion and internal friction angles through different mechanisms. Freeze-thaw cycles primarily influence soil cohesion and internal friction angle by altering the internal structure of soil. On the other hand, particle size characteristics affect these properties through interparticle friction.
3. The interaction between the number of freeze-thaw cycles and soil particle size has a significant impact on soil cohesion and internal friction angle. Under different particle size characteristics, soil cohesion and internal friction angle exhibit complex surface responses to changes in the number of freeze-thaw cycles. Prior to freeze-thaw treatment, soil cohesion generally increases with increasing particle size. After 30 freeze-thaw cycles, as particle size increases, cohesion first increases and then decreases. In contrast, the soil internal friction angle generally shows a downward trend: d_5–10_ mm soil is most affected by freeze-thaw action, while d < 0.25 mm soil is least affected by such action.

This study utilized only one type of soil sample, with freeze-thaw cycle tests confined to a specific temperature range. Consequently, the applicability of the research findings is correspondingly limited. Future studies could expand to multiple soil types and establish a broader freeze-thaw temperature gradient, thereby further enhancing the generalizability of the results and their engineering reference value.

## Data Availability

The datasets generated during and/or analysed during the current study are available from the corresponding author on reasonable request.

## Declarations

### Soil sampling permit statement

Soil samples were collected at Heshan Farm, Nenjiang City, Heilongjiang Province (core area of the black soil region in Northeast China). Prior to sampling, we obtained formal permission from the administrative authority of Heshan Farm. The sampling process complied with the Regulations on the Protection and Utilization of Black Soil in Heilongjiang Province and did not involve any illegal collection or use of black soil.
